# Addressing Hospital Overwhelm During the COVID-19 Pandemic by Using a Primary Health Care–Based Integrated Health System: Modeling Study

**DOI:** 10.2196/54355

**Published:** 2024-06-03

**Authors:** Jiaoling Huang, Ying Qian, Yuge Yan, Hong Liang, Laijun Zhao

**Affiliations:** 1 School of Public Health, Shanghai Jiao Tong University School of Medicine, Shanghai, China; 2 Business School, University of Shanghai for Science and Technology, Shanghai, China; 3 School of Intelligent Emergency Management, University of Shanghai for Science and Technology, Shanghai, China; 4 School of Social Development and Public Policy, Fudan University, Shanghai, China

**Keywords:** hospital overwhelm, primary health care, modeling study, policy mix, pandemic, model, simulation, simulations, integrated, health system, hospital, hospitals, management, service, services, health systems, develop, development, bed, beds, overwhelm, death, deaths, mortality, primary care

## Abstract

**Background:**

After strict COVID-19–related restrictions were lifted, health systems globally were overwhelmed. Much has been discussed about how health systems could better prepare for future pandemics; however, primary health care (PHC) has been largely ignored.

**Objective:**

We aimed to investigate what combined policies PHC could apply to strengthen the health care system via a bottom-up approach, so as to better respond to a public health emergency.

**Methods:**

We developed a system dynamics model to replicate Shanghai’s response when COVID-19–related restrictions were lifted. We then simulated an alternative PHC-based integrated health system and tested the following three interventions: first contact in PHC with telemedicine services, recommendation to secondary care, and return to PHC for recovery.

**Results:**

The simulation results showed that each selected intervention could alleviate hospital overwhelm. Increasing the rate of first contact in PHC with telemedicine increased hospital bed availability by 6% to 12% and reduced the cumulative number of deaths by 35%. More precise recommendations had a limited impact on hospital overwhelm (<1%), but the simulation results showed that underrecommendation (rate: 80%) would result in a 19% increase in cumulative deaths. Increasing the rate of return to PHC from 5% to 20% improved hospital bed availability by 6% to 16% and reduced the cumulative number of deaths by 46%. Moreover, combining all 3 interventions had a multiplier effect; bed availability increased by 683%, and the cumulative number of deaths dropped by 75%.

**Conclusions:**

Rather than focusing on the allocation of medical resources in secondary care, we determined that an optimal PHC-based integrated strategy would be to have a 60% rate of first contact in PHC, a 110% recommendation rate, and a 20% rate of return to PHC. This could increase health system resilience during public health emergencies.

## Introduction

The World Health Organization (WHO) announced the end of the COVID-19 public health emergency of international concern on May 5, 2023. Over the past 3 years, the COVID-19 epidemic has resulted in more than 765 million infections and 6.92 million deaths globally and has involved ongoing outbreaks, infection control via restrictions, the lifting of restrictions, and large-scale infections [1]. The limitations of health care systems worldwide regarding the response to mass infections and admissions have been exposed, and these limitations exist in countries classified as high-performing and resilient countries, as well as in resource-limited countries [2-4]. Although most governments have prudently considered the appropriate time to relax restriction policies, health care systems have unavoidably been overwhelmed, and some even collapsed once restrictions were lifted [5].

Much has been discussed regarding how health care systems could have better prepared for COVID-19 and how to prepare for future pandemics. Topics of discussion include adequate health care workforces and facilities [6], better intensive care unit capacity [7], early intervention to avoid local transmission [8], and the broader application of telemedicine [9]. Some scholars have advocated for the integration and coordination of the health system, including public health and clinical medicine. The role of primary health care (PHC) in COVID-19 management has received attention [10-13], but this attention is obviously insufficient when compared with the attention given to the professional treatment capabilities of large hospitals. In particular, there is a lack of empirical research on the role of PHC. After touring 5 cities in China, the WHO provided recommendations that were predominantly focused on secondary care and epidemiological tracking and control, and the role of PHC was missed again [14].

Distinguishing itself from secondary care for specialist treatment, PHC is regarded as the most inclusive, effective, and efficient approach to enhancing people’s physical and mental health. PHC has great value in a strong, coordinated response to a public health crisis [10,15]. Recent studies show that a strong PHC foundation could effectively mitigate an epidemic. One such case is that of Singapore, which promptly instituted aggressive containment measures by establishing public health preparedness clinics that were supported in a sustained manner by the PHC network [16]. In contrast, PHC resources in the African Union are exceedingly scarce, which resulted in insufficient engagement when dealing with COVID-19 [17-20]. Even in countries with adequate PHC resources, such as the United Kingdom, the health system did not respond quickly and struggled to meet medical demands under a large-scale epidemic [21]. Legido-Quigley and colleagues [7] argued that well-developed integration was a key factor of services influencing resilience during the COVID-19 pandemic in high-performing health systems. Prompt communication and coordination among PHC, public health, and secondary care are essential [22].

At the end of 2022, COVID-19–related restrictions were lifted in China, and an epidemic wave caused by the highly transmissible Omicron SARS-CoV-2 variant placed health services in the country under extreme pressure, especially in metropolises. In Shanghai, which is the most populous metropolis in China and has a permanent population of 25 million, it is extremely difficult to deal with the spread of epidemic infections. At the end of 2022, Shanghai adopted an expansion strategy that involved allocating medical resources in secondary care institutions in a manner that favored patients with SARS-CoV-2 infection. At the same time, Shanghai, as a pilot city, was one of the first cities to promote a hierarchical diagnosis and treatment system based on PHC. As such, Shanghai provides an extremely rare opportunity to explore how the health system of a metropolis can actively respond to large-scale infections, as well as the key role of PHC in this system. In this study, we simulated the large-scale infections that occurred in Shanghai at the end of 2022 by using a simulated environment, wherein we reproduced Shanghai’s response to the challenges of the fast-spreading epidemic. We then tested an alternative strategy that used a PHC-based integrated health system.

## Methods

### Ethical Considerations

Ethics board review was not required, as this study only involved modeling and simulations. All modeling data came from public sources or published papers and did not involve ethical issues.

### Study Design

System dynamics was applied in this study to simulate the mass infections and the health system performance in Shanghai. System dynamics models are established based on the feedback structures (loops) centered around the issue of concern [23,24]. The nonlinear dynamic behaviors derived from these feedback loops shed light on the underlying mechanisms that generate problematic system behaviors, which helps with understanding complex systems and finding fundamental solutions [25]. The use of system dynamics models is a suitable method for investigating public health issues that feature high-complexity systems [26,27]. In recent years, system dynamics has been widely used to model issues related to COVID-19 [28-31].

**Figure 1.**
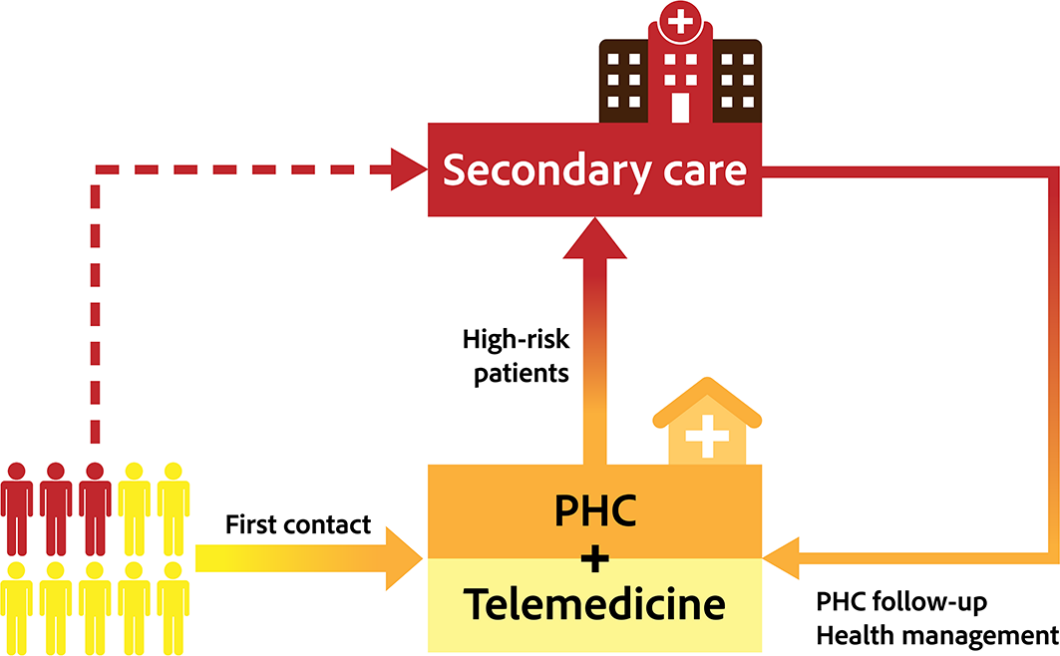
PHC-based health system. PHC: primary health care.

We developed a system dynamics–based model to replicate the health system in Shanghai after COVID-19–related policies were lifted. The following indicators of an overwhelmed health system were used: physician availability (the percentage of patients arriving at the hospital who could be treated) and bed availability (the percentage of patients needing hospitalization who could be admitted) in secondary hospitals. Shanghai’s response to the soaring medical demands was to reallocate medical resources from other divisions to increase the supply of hospital physicians and beds for patients with COVID-19. This policy increased the capacity of secondary hospitals such that more patients could be treated and hospitalized. We also used the system dynamics model to establish a PHC-based integrated health system as an alternative option for addressing hospital overwhelm. The following three critical policy interventions were tested: first contact in PHC, identification of high-risk patients and recommendation to secondary care hospitals, and referral for a return to PHC for follow-up and recovery at the community level (Figure 1). Telemedicine services were also considered in PHC, with which more first contacts could be handled and the capacity of PHC to handle patients could be increased.

### Model Structure

**Figure 2.**
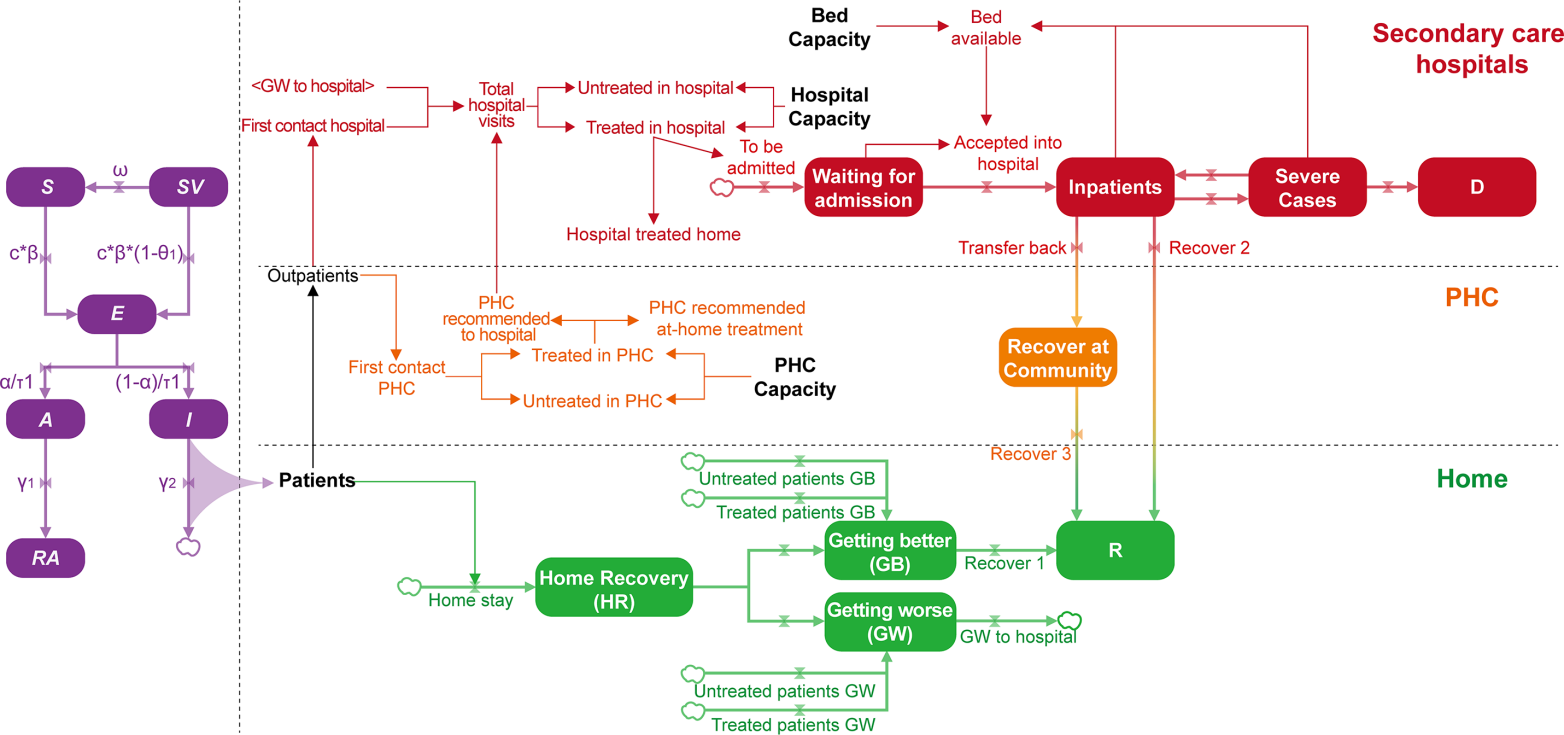
Shanghai system dynamics model of a PHC-based integrated system. *A*: infected population without symptoms; D: death; *E*: infected population during the incubation period; GB: getting better; GW: getting worse; HR: home recovery; *I*: infected population with symptoms; PHC: primary health care; R: recover; *RA*: population in *A* that has recovered; *S*: susceptible population without vaccination; *SV*: susceptible population with vaccination.

The Shanghai model includes the following two parts: the epidemic dynamics representing the mass infections that occurred when COVID-19 restrictions were lifted in Shanghai and the health care system response, as shown in Figure 2.

In Figure 2, the left part depicts an extension of the traditional Susceptible-Exposed-Infectious-Removed model, which we used to model the spread of COVID-19 in Shanghai and compute the number of symptomatic cases, of which a large proportion would require medical services. We disaggregated the total population into the following six groups.

*SV* and *S* represent the susceptible population with vaccination and the susceptible population without vaccination, respectively. The transformation of *SV* to *S* represents the waning effectiveness of COVID-19 vaccines, where ω is the waning effect of vaccination.

*E* represents the infected population during the incubation period. The transformation of *SV* to *E* and *S* to *E* represents the spread of the virus, where c is the contact rate, *β* is the transmission probability, and θ_1_ is the effectiveness rate of vaccination against infection.

*I* and *A* represent the infected population with symptoms and the infected population without symptoms, respectively. α is the percentage of asymptomatic cases, and τ is the incubation period.

*RA* represents the population in *A* that has recovered. γ_1_ is the recovery fraction among asymptomatic cases.

Patients with symptoms (ie, those from population *I*) link the left and right parts of the model. Some patients will recover at home, and others will visit a physician. Among those visiting a physician, some will first contact a PHC institution, while others will contact a secondary hospital directly. PHC institutions and secondary hospitals each have a specific capacity, and when this capacity is reached, new, excess patients cannot be treated and will have to return home. With regard to patients treated in PHC institutions and secondary hospitals, those with mild symptoms will be given prescriptions and sent home to recover. With regard to patients needing further treatment when presenting at the PHC level, general practitioners will recommend them to a secondary hospital; some patients will be hospitalized and become inpatients if hospital beds are available. Over time, some inpatients will recover, whereas others will develop severe illness and eventually recover or die. Patients who recover at home will either improve or worsen, as will untreated and treated patients from PHC institutions and secondary hospitals. The proportion of patients whose condition worsens is highest for untreated patients and lowest for treated patients. Some recovering inpatients in secondary care hospitals might recover at the community level if PHC can provide follow-up health management services. The model equations and parameter settings are detailed in sections 1 and 2 in Multimedia Appendix 1.

### Data Source and Model Validation

We previously developed and validated a model of reopening in Shanghai that accounts for the epidemiological dynamics of the first Omicron wave in this metropolis during the first half of 2022 [32]. The model we established in this study was based on that previous model and was used to simulate the second Omicron wave, specifically the time when most intervention prevention control measures were lifted at the end of 2022. Data related to the spread of Omicron in Shanghai, such as the contact rate, transmission possibility, asymptomatic rate, incubation period, and recovery fraction, were obtained from previous literature about COVID-19 and, especially, Omicron (further details are reported in section 2 and Table S1 in Multimedia Appendix 1). Data related to individuals’ behaviors, such as the rate of first contact in PHC and the rate of recovery at home, were set according to estimations based on our investigation of PHC, hospitals, and the community. Sensitivity tests for these parameters were conducted to check the robustness of the model (section 3.2 in Multimedia Appendix 1).

The validation of model behavior usually involves the comparison of simulation results with real-world data. Because mass COVID-19 testing was no longer required, accurate infection data were no longer available; news reports on viral infections and the level of pressure on medical resources were used as references. The model results revealed patterns that were similar to the actual situation during the second Omicron wave in Shanghai, thereby confirming the validity of the model (further details are reported in section 3 in Multimedia Appendix 1). As a result, we determined that this model could provide a simulated environment to facilitate the exploration of effective policies regarding the response to mass infections in a metropolis.

## Results

### Scenario 1: Medical Resource Reallocation in Secondary Care

**Figure 3.**
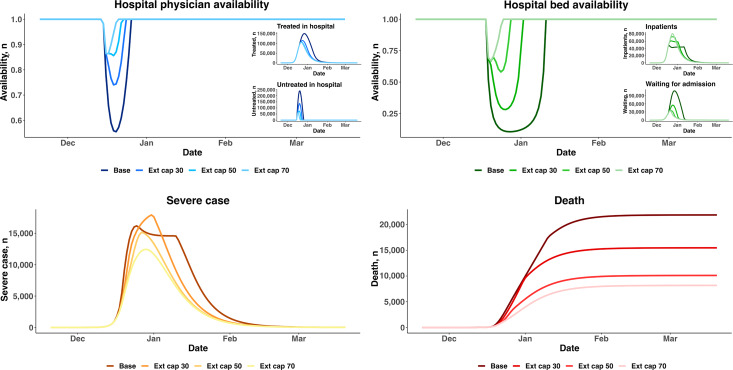
Expanding capacity to meet soaring demand. Base: baseline; Ext cap 30: 30% capacity extension; Ext cap 50: 50% capacity extension; Ext cap 70: 70% capacity extension.

When the strict intervention prevention controls were lifted, the policy focus changed from preventing the spread of COVID-19 to the timely treatment of patients with COVID-19. When facing massive increases in infections, physicians’ availability could decline to as low as 55% if no interventions were adopted. In the case of Shanghai, a series of measures was taken to deal with the impact of large-scale infections on hospitals. When hospital physician capacity and hospital bed capacity were increased by 70% of the original capacities, the lowest physician availability and bed availability changed to approximately 85% and 70%, respectively. Moreover, bed shortages lasted approximately 8 days, which was around one-third of the bed shortage time for the scenario with no capacity extension. The peak number of severe cases decreased, as more patients could be treated promptly. Consequently, the cumulative number of deaths decreased to less than half of that for the scenario without additional resources, as shown in Figure 3.

### Scenario 2: PHC-Based Integrated Health Care System

#### Scenario 2.1: Increasing the Rate of First Contact in PHC Plus PHC Telemedicine

**Figure 4.**
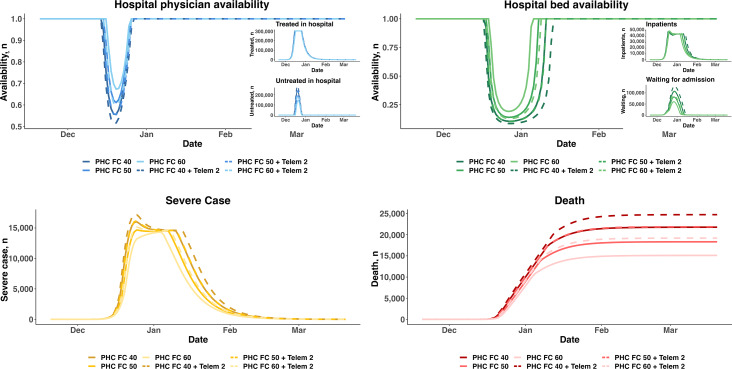
Scenarios with various rates of first contacts in primary health care with and without telemedicine. PHC FC 40: 40% rate of first contact in primary health care without telemedicine; PHC FC 50: 50% rate of first contact in primary health care without telemedicine; PHC FC 60: 60% rate of first contact in primary health care without telemedicine; PHC FC 40 + Telem 2: 40% rate of first contact in primary health care with telemedicine; PHC FC 50 + Telem 2: 50% rate of first contact in primary health care with telemedicine; PHC FC 60 + Telem 2: 60% rate of first contact in primary health care with telemedicine.

Facing huge increases in the number of patients, we examined ways to increase the rate of first contact in PHC, in which PHC telemedicine services were also considered. Under such circumstances, 6 scenarios were simulated, with the rate of first contact in PHC with and without telemedicine services set to 40%, 50%, and 60%. Figure 4 shows that replacing the worst scenario (40% rate of first contact in PHC without telemedicine) with the best scenario (60% rate of first contact in PHC with telemedicine) increased the lowest level of secondary hospital physician availability and that of secondary hospital bed availability by 32% (from 51% to 67%) and 111% (from 9% to 19%), respectively. Moreover, the duration of bed shortages dropped from approximately 30 days to approximately 20 days. Because more patients were promptly treated in the best scenario, the number of cumulative deaths decreased from 24,740 in the worst scenario to 15,837—a 56% decrease.

#### Scenario 2.2: Referral Recommendation Rate for High-Risk Patients

**Figure 5.**
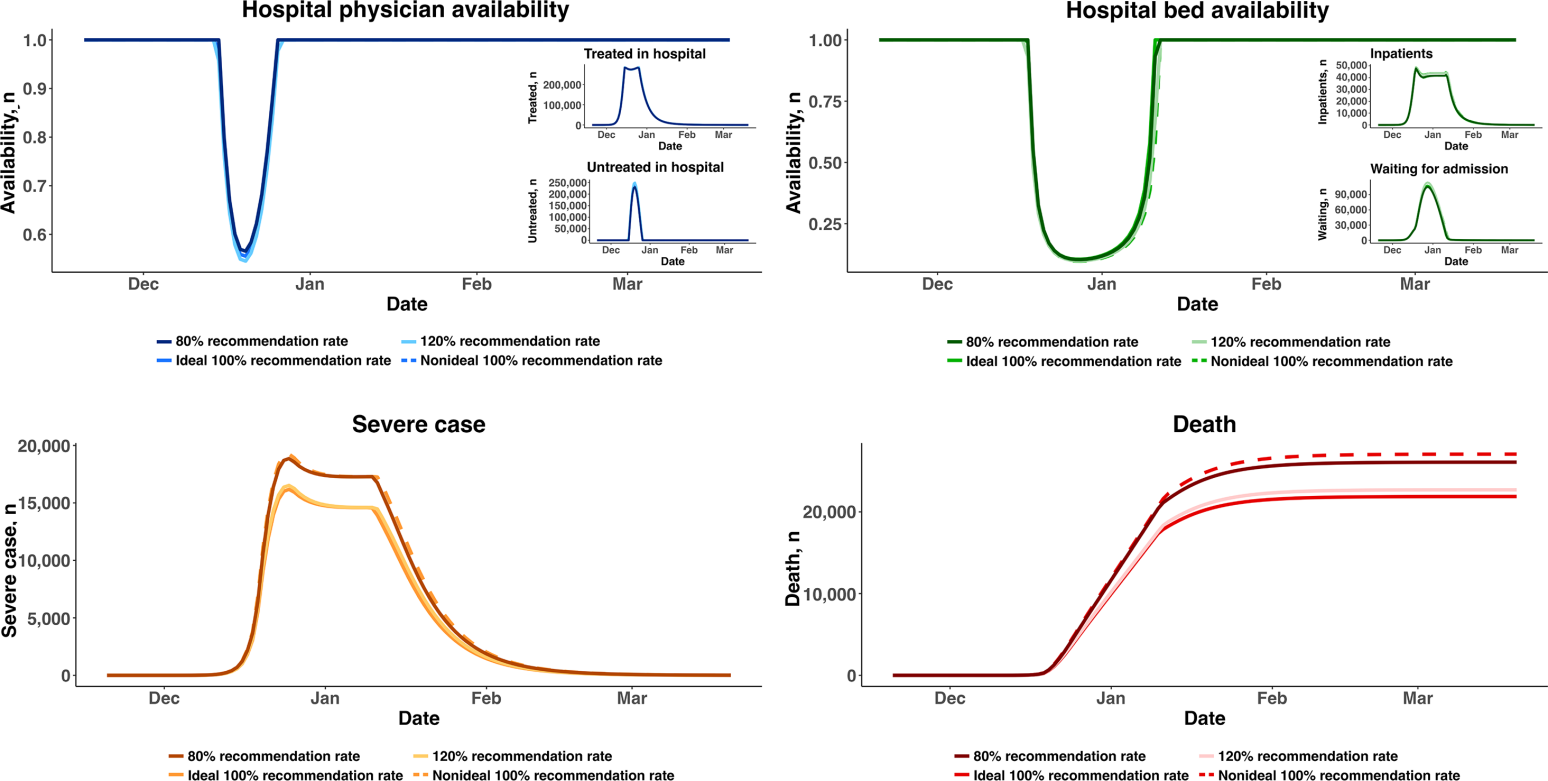
Scenarios involving general practitioners in primary health care with differing capabilities for identifying high-risk patients. “Ideal” refers to no underrecommendation and no overrecommendation. “Nonideal” refers to underrecommendation and overrecommendation happening at the same time.

Two of the main tasks of general practitioners in PHC are to identify high-risk patients and provide referral recommendations to secondary care, which are related to the professional capabilities of general practitioners. We simulated the following four scenarios: (1) a recommendation rate of 80%, meaning that 20% of patients requiring advanced treatment in a hospital were not identified (ie, underrecommendation); (2) a recommendation rate of 100% without underrecommendation or overrecommendation, which is an ideal scenario; (3) a recommendation rate of 120%, meaning that 20% of patients were overreferred to secondary care; and (4) a recommendation rate of 100% but with underrecommendation and overrecommendation happening at the same time. Underrecommendation and overrecommendation, respectively, slightly increased and decreased the physician availability and bed availability. However, with underrecommendation, in which 20% of patients who needed to be treated in a hospital were not referred, some patients developed severe illness due to improper treatment, leading to more severe cases and more cumulative deaths, as shown in Figure 5.

#### Scenario 2.3: Follow-Up Health Management Services in PHC

**Figure 6.**
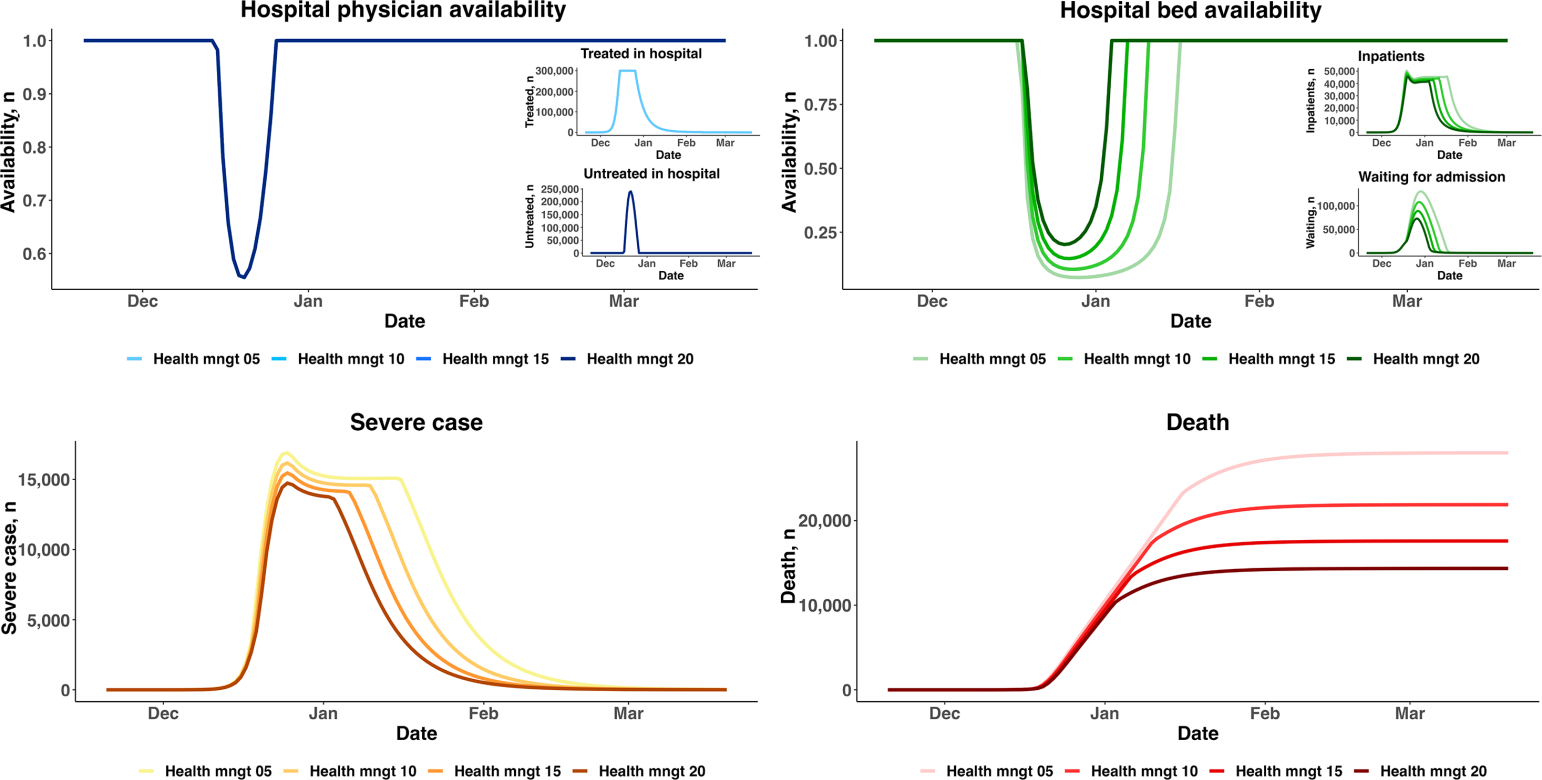
Scenarios with different percentages of patients returning to primary health care for recovery. Health mngt 05: 5% rate of health management in the community; Health mngt 10: 10% rate of health management in the community; Health mngt 15: 15% rate of health management in the community; Health mngt 20: 20% rate of health management in the community.

When facing bed shortages, some patients with mild symptoms who are nearly recovered can be transferred to recover in the community if PHC can provide follow-up health management services. We simulated four scenarios under which 5%, 10%, 15%, and 20% of hospital inpatients were transferred back to PHC to recover. As shown in Figure 6, physician availability was not affected, but hospital bed availability improved. When 20% of inpatients could recover in the community, the lowest bed availability level reached approximately 20%, whereas this level reached 7% in the scenario where only 5% of inpatients were referred to PHC to recover. Moreover, the number of days with bed shortages was nearly halved. As a result, the peak number of severe cases decreased from 16,897 to 14,737, and the cumulative number of deaths dropped from 28,008 to 14,344.

#### Scenario 2.4: Mixed Policy Interventions

**Figure 7.**
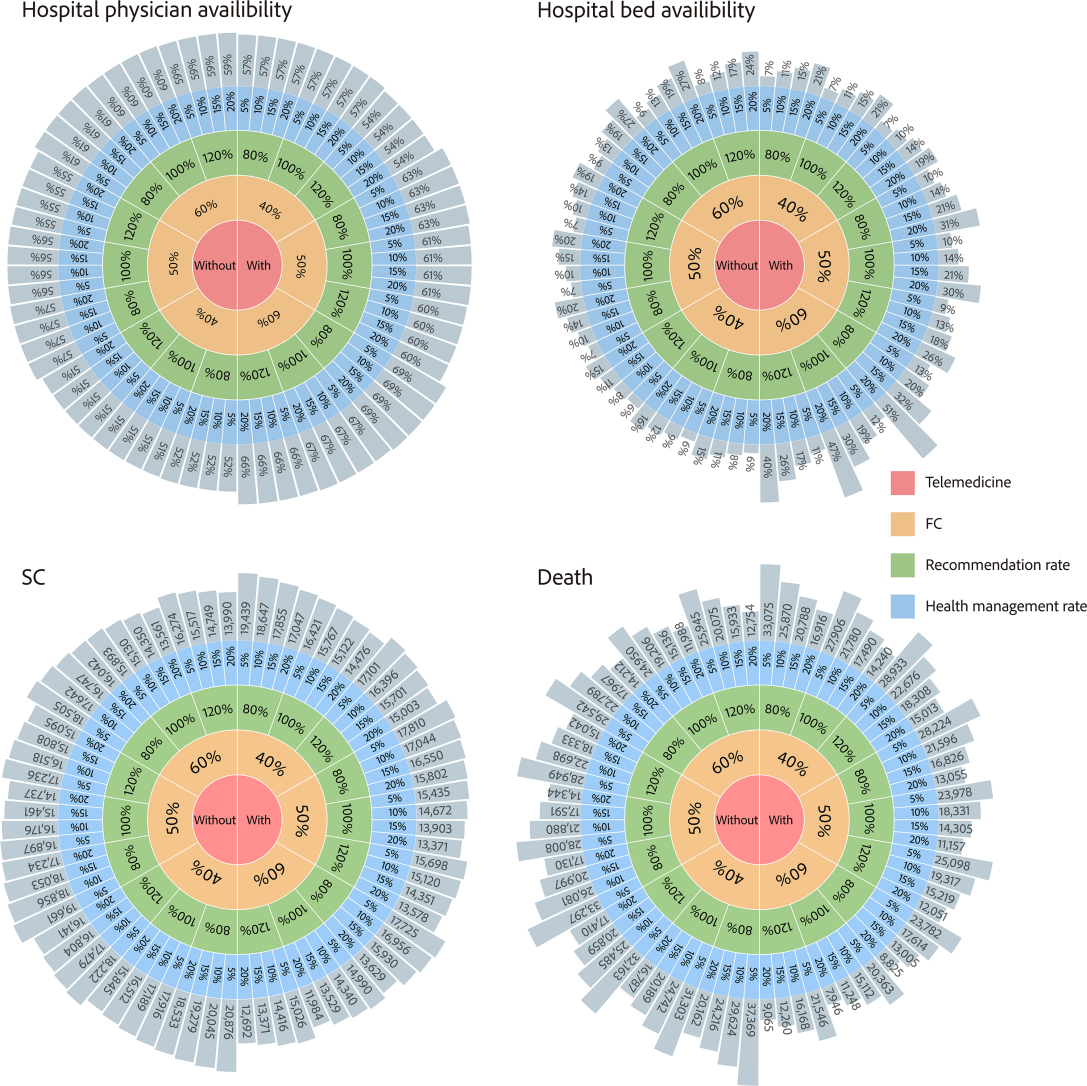
Overall impact of integrated primary health care (PHC). The red, orange, green, and blue areas represent PHC with or without telemedicine, the rate of FC in PHC, the rate of PHC recommendation to secondary care, and the rate of referral to return to PHC, respectively. The gray areas represent the biggest medical resource gaps, including hospital physician availability and hospital bed availability, and the resulting peak number of SCs and cumulative deaths. FC: first contact; SC: severe case.

We integrated the above three policy interventions to investigate the overall impact of a PHC-based system, as shown in Figure 7. The model simulation results showed that the lowest level of hospital physician availability ranged from 51% in the worst case (40% rate of first contact in PHC without telemedicine) to 69% in the best case (60% rate of first contact in PHC with telemedicine). The lowest level of hospital bed availability varied even more, ranging from 6% in the worst case (40% rate of first contact in PHC without telemedicine and 5% rate of health management in the community) to 51% in the best case (60% rate of first contact in PHC with telemedicine, recommendation rate of 80%, and 20% rate of health management in the community). With regard to the number of severe cases, the worst case peaked at 20,876 severe cases (40% rate of first contact in PHC without telemedicine, underrecommendation rate of 20%, and 5% rate of health management in the community), and the best case peaked at 11,984 severe cases (60% rate of first contact in PHC with telemedicine, recommendation rate of 100%, and 20% rate of health management in the community)—a decrease of approximately 75%. As for cumulative deaths, the worst case was 37,369 deaths, and the best case was only 7946 deaths—a 79% reduction. Furthermore, we identified the following relatively optimal policy intervention mix: a 60% rate of first contact in PHC with telemedicine services, a 20% rate of referral to return to PHC, and a recommendation rate of 100% to 120%.

## Discussion

### Principal Findings

The experience of hospital overwhelm during the COVID-19 pandemic highlights the need to reflect on existing health systems and search for more proactive solutions during an epidemic. In this study, we constructed a simulated policy environment to replicate Shanghai’s response to the mass SARS-CoV-2 infections that occurred once restrictions were lifted. Specifically, the Shanghai Municipal Health Commission deployed medical resources in secondary care hospitals, in a manner that favored patients with SARS-CoV-2 infection, as quickly as possible. This strategy included increasing the availability of beds and reallocating more medical staff from other departments to promptly treat critically ill patients and prevent deaths. This efficient and decisive response allowed Shanghai to avoid the large-scale congestion and overwhelm of the health care system. However, Shanghai’s strategy worked under the assumptions that advanced medical resources would be available and could be deployed, and the strategy largely depended on the government’s strong decision-making and coordination capabilities. At the same time, we realize that relying on PHC to alleviate congestion is an important strategy to achieve the effective allocation of medical resources, rather than only relying on temporary expansion in secondary care [33].

We proposed an alternative PHC-based strategy and tested this in a simulated policy simulation environment. We tested the rate of first contact in PHC, the rate of identifying high-risk patients for recommendation to a specialist, and the rate of return to PHC for recovery. According to the simulation results, increasing the rate of first contact in PHC could effectively alleviate the shortage of specialists in large hospitals. Additionally, telemedicine application in PHC contributed substantially to reducing congestion within hospitals engaged in COVID-19 treatment. In our model, a 60% rate of first contact in PHC with telemedicine could increase the lowest level of secondary hospital physician availability from 51% to 67% and reduce the number of cumulative deaths by 9630. The value of first contact in PHC for patients is receiving immediate medical treatment to avoid severe illness or death caused by delays in treatment, as well as reducing the shortage of medical resources in secondary hospitals. COVID-19 has accelerated the development of telemedicine. Alexander and colleagues [34] used a nationally representative audit of outpatient care to characterize primary care delivery in the United States and found that the pandemic was associated with a >25% decrease in primary care volume, which has been offset in part by increases in the delivery of telemedicine. Some believe that the boom in telemedicine during the COVID-19 pandemic could worsen health disparities [35], especially for racial and ethnic minority groups; those living in rural areas; and individuals with limited English proficiency, low literacy, or low income [36]. Nevertheless, telemedicine is an inevitable future developmental trend.

The rate of identifying high-risk patients is a crucial indicator of PHC worker capacity. We found that underidentification could result in more severe illness and more deaths, whereas overidentification could increase congestion in hospitals to some degree. For example, in the scenario with a 50% rate of first contact in PHC with telemedicine, a 120% recommendation rate reduced hospital specialist availability from 61% to 60%, whereas an 80% recommendation rate increased hospital specialist availability from 61% to 63%. A similar impact was observed on hospital bed availability. However, underrecommendation resulted in some patients (ie, those needing further treatment) failing to seek timely medical care and thus higher rates of severe illness and an increase of 3265 cumulative deaths. According to the simulation results, the effect of accurately identifying high-risk patients is limited in the existing system, possibly due to a low rate of first contact in PHC. Unlike countries in Europe and North America, China has a loose medical referral system rather than a strict referral system based on first contact in PHC [37]. China established its PHC system after the new health care reform in 2009 [38]. In October 2016, the Chinese government launched the Healthy China 2030 initiative, in which a critical component is developing a patient referral model [39]. In contrast, gatekeeping systems can ensure the efficient use of scarce medical resources in secondary care; to date, there has been no action plan to enforce the patient referral model [37]. The rate of first contact in PHC has remained at approximately 30% to 50% for the past 10 years. However, in scenarios where PHC first contact–based referral is strictly implemented, such as in the United Kingdom [40], we believe that accurate risk identification in PHC is important.

We also considered the rate of return to PHC for recovery in the community, which can accelerate bed turnover in secondary hospitals. The model simulation results showed that increasing the rate of return to PHC from 5% to 20% would increase bed availability from 6% to 16%, thereby reducing the number of cumulative deaths by approximately 13,000. According to the WHO, referral is a bidirectional process that acknowledges not only the role of the specialist but also the critical role of PHC workers in coordinating patient care over the longer term [41]. In May 2023, Shanghai issued an important document*—Implementation Plan to Further Enhance the City’s Community Health Service Capabilities*—focusing on strengthening 4 functions in community health centers—PHC, health management, rehabilitation, and nursing—as well as the primary public health network [42]. COVID-19 has definitely brought challenges to PHC, but it has also provided new opportunities.

Interestingly, we also found a multiplier effect with combined policy interventions. For example, offering telemedicine services, increasing the rate of first contact in PHC from 40% to 60%, and raising the rate of referral to return to PHC from 5% to 20% respectively increased bed availability by 16.67%, 50%, and 167%. However, when combined, these policies increased the lowest bed availability level by 683%. Optimal policy intervention combinations are widely applied in health, climate change, and economics (eg, funding instruments). Policy mixing implies a focus on trade-off interactions and interdependencies among different policies, as they affect the extent to which the intended policy outcomes are achieved. It provides a window of opportunity to reconsider basic and often hidden assumptions to better deal with complex, multilevel, multiactor realities [43]. In this study, we identified a relatively optimal policy combination (ie, a 60% rate of first contact in PHC, a 110% recommendation rate, and a 20% rate of return to PHC) that could establish a strong PHC foundation and increase health system resilience by reducing medical resource gaps in responding to public health emergencies. The interplay of policies and instruments, as well as the deliberate design of policy mixes and portfolios of interventions, has received surprisingly little practical and theoretical attention so far and is vastly underrated [44].

Using the scenario of reopening in Shanghai, we built a health care system for metropolises to deal with large-scale infections and verified the role of PHC through a system simulation model. However, our study has some limitations. First, real-world data were missed, especially epidemiological data, as mass COVID-19 testing was canceled. We validated our model based on information from news reports indicating the development of the Omicron wave and web-based information. Second, data related to individuals’ behaviors, such as the rate of first contact in PHC and the rate of recovery at home, are not available. We estimated these parameters according to our investigation of PHC institutions, hospitals, and communities. Third, this study simulated a PHC-based integrated health system responding to large-scale infections (including parameters such as first-contact rate, referral rate, and recovery fraction), but our models did not tell us how the integrated system could be improved. More attention should be paid to integrated health systems in the near future by conducting more case studies or implementation research.

### Conclusions

Rather than focusing on secondary care, in this study, we proposed an alternative—strengthening the health system via a bottom-up approach by using PHC as a foundation to better respond to a public health emergency. Per our PHC-based health system, an optimal PHC-based integrated strategy would be to have a 60% rate of first contact in PHC, a 110% recommendation rate, and a 20% rate of return to PHC, which could increase health system resilience during public health emergencies. A robust PHC-based integrated health system, in addition to the temporary deployment of medical resources in secondary care, could maximize the use of limited medical resources to actively respond to large-scale increases in infections. This study provides an optimal solution for constructing a PHC-based integrated health system to respond to large-scale infections. We acknowledge that there is a long way to go to achieve an integrated health system, as getting PHC to communicate and interact seamlessly with secondary care is extremely challenging globally. We advocate increasing investments in PHC to promote its overall development and conducting more research on integrated health systems in the near future.

## Supplementary material

10.2196/54355 Multimedia Appendix 1 Model description and definitions, parameter settings, and model validation.

## Abbreviations

PHC: primary health care
WHO: World Health Organization
