# Supplementary material for: Addressing Hospital Overwhelm During the COVID-19 Pandemic by Using a Primary Health Care–Based Integrated Health System: Modeling Study
Source: JMIR Med Inform. 2024 Jun 3;12:e54355. doi: 10.2196/54355 (PMC11185287; doi:10.2196/54355)
Supplement: Multimedia Appendix 1 [file medinform-v12-e54355-s001.docx]

# Model Description and Definitions

In this section, we provide a detailed description of our model, including model structure, equations, and parameter setting procedures.

The model includes basically two parts, 1) the spread of the COVID-19 and 2) the medical demand derived from the infection of the disease and the medical supply from the healthcare system in Shanghai.

The first part of the model an extension of the classic SEIR model, which computes the spread of the COVID-19 and the asymptomatic cases and symptomatic cases, a large part of which require medical service, as shown in Figure S1.

$$V_{SV\to E}$$

$$V_{S\to E}$$

$$V_{SV\to S}$$

$$V_{E\to A}$$

$$V_{E\to I}$$

$$V_{A\to RA}$$

patiencts


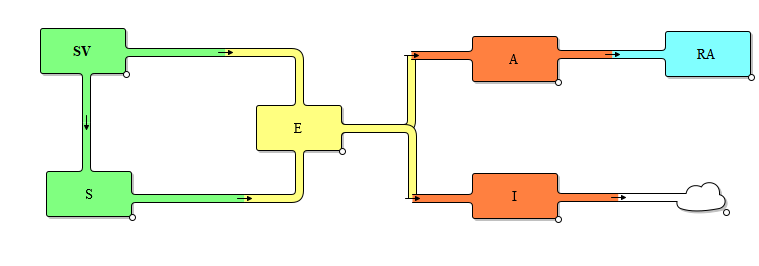


Figure S1. The first part of Shanghai model

The total population are divided into the following groups:

**SV** represented the vaccinated susceptible population;

**S** represented the susceptible population without vaccination;

**E** represented the exposed population in the incubation period;

**A** represented the infected population without symptoms;

**I** represented the infected population with symptoms;

**R_A_** represented the recovered population without symptomatic.

**E**, **I** and **A** can all transmit the disease when contacting with **S** or **SV**. Thus, the spread of the COVID-19 can be represented by the following equations:

$$V_{SV\to E}=(E+I+A)*SV/N*c*\beta*(1-\theta_{1})$$

$$V_{S\to E}=\left( E+I+A \right)*S/N*c*\beta$$

where c is the contact rate, β is the transmission probability, and θ_1_ is the vaccination effectiveness (VE) against infection.

Researches have proved that the protection provided by vaccination wan overtime ^[1-2]^. Thus,

$V_{SV\to S}=SV*$ω

where ω as the waning fraction.

**E** will develop into asymptomatic cases or symptoms cases after the incubation period. Thus

$$V_{E\to A}=\frac{\alpha*E}{\tau_{1}} V_{E\to I}=\frac{\left( 1-\alpha\right)*E}{\tau_{1}}$$

where α is the percentage of asymptomatic cases and τ_1_ is the incubation period.

**A** do not need any treatment and will recover (here recover means do not infect others anymore) over time. Thus,

$$V_{A\to RA}=A*\gamma_{1}$$

where γ_1_ is the recovery fraction of asymptomatic cases, which is the reciprocal of the recovery period.

**I** need treatment, be it to rest at home or to visit doctor. Thus,

$$patients=I*\gamma_{2}$$

where γ_2_ is the treatment fraction of symptomatic cases, which is the reciprocal of the time patients take actions to treat COVID-19.

In summary, the spread of the COVID-19 can be presented by the following differential equations:

$$\frac{dSV}{dt}=-SV* \omega-\left( E+I+A \right)*\frac{SV}{N}*c*\beta*(1-\theta_{1})$$

$$\frac{dS}{dt}=SV* \omega-(E+I+A)*S/N*c*\beta$$

$$\frac{dE}{dt}=\left( E+I+A \right)*\frac{SV(1-\theta_{1})+S}{N}*c*\beta-\frac{E}{\tau_{1}}$$

$$\frac{dA}{dt}=\frac{\alpha*E}{\tau_{1}}-A*\gamma_{1}$$

$$\frac{dI}{dt}=\frac{(1-\alpha)*E}{\tau_{1}}-I*\gamma_{2}$$

$$\frac{dR_{A}}{dt}=A*\gamma_{1}$$

The second part of the model represents the medical demand from the spread of COVID-19, which is related to V1, and the medical supply from the healthcare system in Shanghai, which include primary healthcare (PHC) and secondary hospitals (Hos). Therefore, the gap for medical service could be calculated, which affect the number of severe cases and cumulative death, as shown in Figure S2.


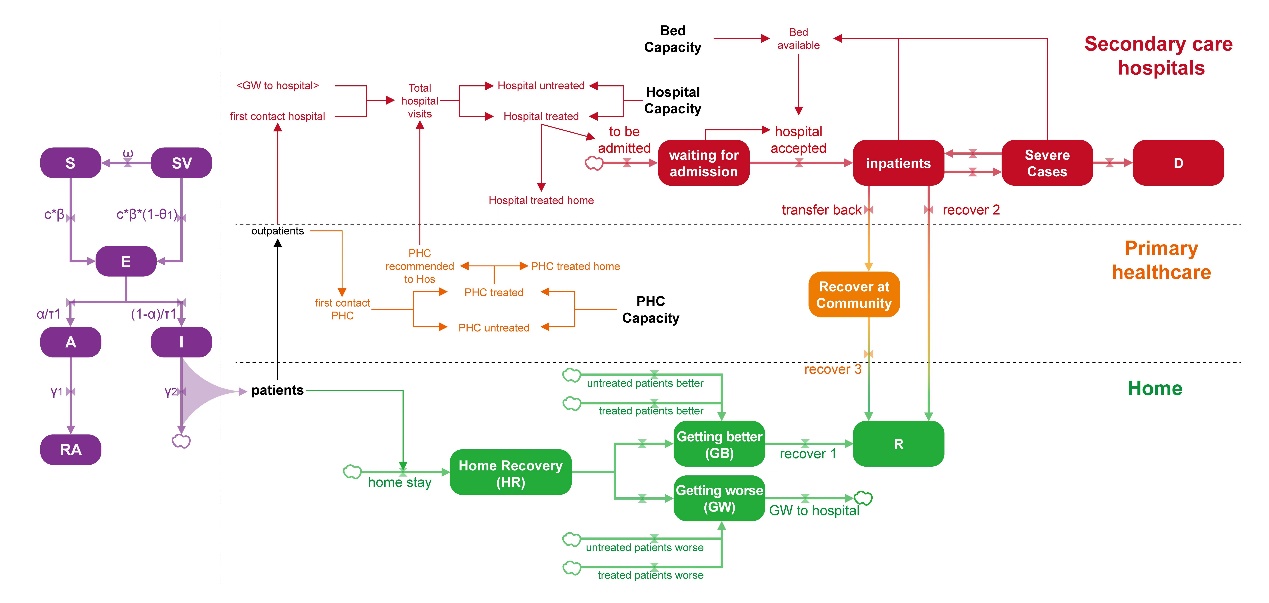


(V13)

(V12)

(V11)

(V10)

(V9)

(V8)

(V7)

(V5)

(V6)

(V4)

(V3)

(V1)

(V2)

Figure S2. The second part of Shanghai model

**HR** reflected the infected population with symptoms that recover at home;

**GB** and **GW** reflected those people recover at home (RH) who get better and get worse respectively;

**R** represented the population recovered;

**RAC** represented the population recovering at community level.

**WA (waiting for admission)** represented the patients that to be accepted into hospitals;

**INP (inpatients)** represented the patients accepted into hospitals, which became inpatients;

**SC (Severe Cases)** represented the inpatients developed into severe cases;

**D** represented the death population;

Of all the *patients* that need treatment, a percent would rest at home for recovery (V1) and the other (1-а) percent would require a visit to doctors (V2) (0<а<1). Thus,

$$V1=a*patients V2=(1-a)*patients$$

The patients choose to recover at home (V1) would accumulate in the stock **HR**. Over the observation time, $\tau_{2}$, b of **HR** would get better and other (1-b) of **HR** would get worse (0<b<1). Thus,

$$V_{RH\to GB}=\frac{b*RH}{\tau_{2}} V_{RH\to GW}=\frac{\left( 1-b \right)*RH}{\tau_{2}}$$

For those get better, they would recover at a rate of γ_3_ and for those get worse, they would visit doctor later at a rate of 1/τ_3_, where τ_3_ is the react time. Thus,

$$recover 1=GB*\gamma_{3} GW to hospital=\frac{\mathrm{GW}}{\tau_{3}}$$

For those visit doctors (V2), d of them would first contact PHC (V3) while the other (1-d) would first contact doctors (V4) at secondary care hospitals(0<d<1). Thus,

$V3=d*V2$ $V4=(1-d)*V2$

PHC has its capacity (C_PHC_). If patients visit PHC (V3) is above the capacity, they will not be able to treated, and have to go back to home (V5). Otherwise, the patients will be treated (V6). Thus,

$$V5=\max\left( 0, V3-C_{PHC} \right) V6=min(V3,C_{PHC})$$

For those treated patients (V6), e of them will be given subscriptions and go back to home for recovery (V7), while (1-e) of those will be recommended to go to secondary care (V8) for further medical check and treatment (0<e<1). Thus,

$$V7=e*\sigma*V6 V8=\left( 1-e*\sigma\right)*V6$$

where σ is family doctor’s ability to identify high risk patients and recommend them to hospitals for further medical treatment.

The patients visit secondary care hospitals (V9) include three parts, those who choose to first contact at hospital V4, those who are recommended to hospital from V8 and those people who get worse during their home recovery and have to go to visit hospital doctors.

$$V9=V4+V8+GW to hospital$$

Similar to PHC, Hospital has its capacity (C_HOS_). They can only treat patients within their capacity (V10). If patients are over hospital capacity, they are not able to visit doctor but have to go back to home without treatment (V11). Thus,

$$V10=\min\left( V5, C_{Hos} \right) V11=\max\left( 0, V5-C_{Hos} \right)$$

After treatment (V10), f of V10 would be given subscriptions to recover at home (V12) and other (1-f) of V10 would need to be hospitalized (V13) (0<f<1). Thus,

$$V12=f*V10 V13=(1-f)*V10$$

Those need to be hospitalized will accumulate in the stock WA. Hospital bed also has its capacity, C_Bed_. When beds are occupied by inpatients and severe cases, they are not available to accept new patients. Therefore, only the available beds (C_Bed_ – INP – SC) can receive patients. Thus,

$$V_{WA\to INP}=min(WA, C_{Bed}-INP-SC)$$

Some INP will develop into severe cases. Thus,

$$V_{INP\to SC}=\frac{\eta*INP*(1-Ve*\theta_{2})}{xyz}$$

where η is the fraction that develop into SC, θ_2_ is the vaccination effectiveness against SC, *x* is the effect of PHC competency to correctly recommend the high-risk patients to hospital, *y* is the effect of bed availability, and *z* is the effect of hospital doctor availability.

SC would back to INP or death. Thus,

$$V_{SC\to INP}=SC/\tau_{4}V_{D}=\frac{\delta*SC*(1-Ve*\theta_{3})}{y}$$

where $\tau_{4}$ is the time for ICU stay, δ is the fraction of death, θ_3_ is the vaccination effectiveness against death.

Some INP would recovery at hospital or transfer back to recover at community if PHC could provide follow-up health management service. Thus,

$$recover 2=INP*\gamma_{4} transfer back=INP*\gamma_{5}$$

where γ_4_ is the reciprocal of the time for hospital duration, γ_5_ is the fraction of inpatients transfer back to community.

Those patients who have been transferred back to PHC for further recovery would accumulate in the stock **RAC**, and gradually recover over time. Thus,

$$recover 3=RAC*\gamma_{6}$$

Where γ_6_ is the fraction of recovery at community.

In summary, the treatment of the symptomatic COVID-19 cases could be presented by the following differential equations:

$$\frac{dRH}{dt}=a*V1-\frac{RH}{\tau_{2}}$$

$$\frac{dGB}{dt}=\frac{b*RH}{\tau_{2}}+p_{1}*\left( V6+V9 \right)+p_{2}*\left( V8+V11 \right)-GB*\gamma_{3}$$

$$\frac{dGW}{dt}=\frac{(1-b)*RH}{\tau_{2}}+(1-p_{1})*\left( V6+V9 \right)+(1-p_{2})*\left( V8+V11 \right)-V13$$

$$\frac{dWA}{dt}=V_{12}-min(WA, C_{Bed}-INP-SC)$$

$$\frac{dINP}{dt}=min\left( WA, C_{Bed}-INP-SC \right)+SC/\tau_{4}-\frac{\eta*INP*\left( 1-Ve*\theta_{2} \right)}{xyz}-INP*(\gamma_{4}+\gamma_{5})$$

$$\frac{dSC}{dt}=\frac{\eta*INP*(1-Ve*\theta_{2})}{xyz}-\frac{\delta*SC*(1-Ve*\theta_{3})}{y}-SC/\tau_{4}$$

$$\frac{dD}{dt}=\frac{\delta*SC*(1-Ve*\theta_{3})}{y}$$

$$\frac{dR_{RAC}}{dt}=INP*\gamma_{5}-RAC*\gamma_{6}$$

$$\frac{dR}{dt}=GB*\gamma_{3}+INP*\gamma_{4}+RAC*\gamma_{6}$$

# Parameter setting

The following table consists all the parameters details, including explanation of its meaning and its value, unit and how the parameter value is set.

Table S1 Model parameter setting

| **Var** | **Explanation** | **Value** | **unit** | **Source** |
| --- | --- | --- | --- | --- |
| $c$ | contact rate: average number of contacts per person per day | 14.8 | Times/day/person | The normal contact rate was 14.8.^3^ |
| $\beta$ | probability of transmission per contact | 0.08 | 1/ times | Transmission probability is closely related to the basic reproduction number R_0_. In this setting, the R_0_ was estimated to be approximately 7, which is higher than the delta variant^4, 5^ |
| $\theta_{1}$ | vaccination effectiveness (VE) against infection | 0.7 | Dmnl (percent) | Based on the research on various local vaccine, the mean value of vaccination effectiveness against infection was about 0.7.^6^ |
| $\omega$ | waning fraction | 1/270 | 1/day | A pooled estimates show that 9 months after completing a primary vaccine cycle, VE against symptomatic infection with the Omicron variant was less than 10%, so the time is set at 9 months.^7^ |
| $\alpha$ | the percentage of asymptomatic cases | 0.3 | % | People with Omicron much more likely to be asymptomatic.^12^ |
| $\tau_{1}$ | the incubation period | 3 | day | Omicron’s incubation period is shorter than the original SARS-CoV-2 (five days or greater) and the Delta variant (four days). Omicron may have an average incubation period of three days.^8,9^ |
| $\gamma_{1}$ | the reciprocal of time for asymptomatic cases to recovery | 0.1 | 1/day | A few existing studies suggest people are unlikely to remain infectious after 10 days.^10^ |
| $\gamma_{2}$ | the reciprocal of the time symptomatic population takes actions to treat COVID-19 | 1 | 1/day | When symptoms are shown, most people will act soon, either rest at home or go to visit doctor. |
| $a$ | the percentage of *I* would rest at home for recovery | 0.5 | Dmnl (percent) | Many people just rest at home when infected but also many people visited doctors. Scenario Setting based on local observation. |
| $\tau_{2}$ | the observation time for home recovery population | 3 | day | Normally when people recover at home would give 2-3 days for observation. |
| $b$ | percentage of HR that get better | 0.8 | Dmnl (percent) | The majority of the people recover at home would self-recover, as Omicron causes mostly mild symptoms. |
| $\tau_{3}$ | the react time: the people who get worse would visit a doctor | 3 | day | Scenario Setting based on local observation. |
| $d$ | percentage of people who visit doctors first contact PHC | 0.5 | Dmnl (percent) | Policy intervention point. Initially set at 50% and various values were simulated in different scenarios. |
| $e$ | the percentage of treated patients recommended to hospital after PHC treatment | 0.1 | Dmnl (percent) | As Omicron causes mostly mild symptoms, the majority of the patients visited PHC could be treated and prescribe medicine to recover at home. Scenario Setting based on local observation. |
| $f$ | the percentage of people who were treated by the doctors need to be hospitalized | 0.05 | Dmnl (percent) | According the data from WHO, high risk population has a hospitalization rate of 6%, moderate risk population has a hospitalization rate of 3% and low risk population has a hospitalization rate of 0.5% only^11^. Consider low risk population probably will recover at home, without visiting secondary hospital, we set this parameter to be 5%. |
| $\gamma_{3}$ | the reciprocal of the time period that those getting better needed to recover | 1/7 | 1/day | Scenario Setting |
| $\gamma_{4}$ | the reciprocal of the time period needed to stay at hospital | 1/7 | 1/day | Normally, if not severe cases, people stay in hospital for 7 days. |
| $\gamma_{5}$ | the fraction of the INP recovery at community with PHC health management | 0.1 | 1/day | Policy intervention point. Initially set at 10% and various values were simulated in different scenarios. |
| $\eta$ | the fraction of inpatients develops into severe cases | 0.035 | 1/day | Scenario Setting |
| $\theta_{2}$ | vaccination effectiveness against severe cases | 0.8 | Dmnl (percent) | The mean value of vaccination effectiveness against SC was about 0.8.^6^ |
| $\tau_{4}$ | the time for ICU stays | 7 | day | Scenario Setting |
| $\delta$ | the fraction of death | 0.03 | 1/day | Scenario Setting |
| $\theta_{3}$ | vaccination effectiveness against death | 0.9 | Dmnl (percent) | The mean value of vaccination effectiveness against death was about 0.9.^6^ |
| $P_{2}$ | the percentage of untreated patients getting better | 0.7 | Dmnl (percent) | Scenario Setting |
| $P_{3}$ | the percentage of treated patients getting better | 0.95 | Dmnl (percent) | Scenario Setting |
| σ | the ability family doctors could identify high risk patients and recommend them to hospital | 1 | Dmnl (percent) | Policy intervention point. Initially set at 100% and various values were simulated in different scenarios. |

# Model validation

**3.1 Comparison of model simulation result with real world situation**

Shanghai gradually relieved the IPC policies for COVID-19 from Nov 2022. The infected population increased fast in Dec and reached peak within two-week time causing huge pressure to medical system. Later, the daily new infection started to reduced and in mid Jan, the hospitals returned to normal. We gathered information about the omicron wave from news reports and information online. Detailed information is shown in Figure S3.

2023.01.08

2023.01.18

Report from Shanghai community: Shanghai has passed the peak of the current outbreak, and the intensity of community transmission has significantly decreased. The number of emergency cases in medical institutions has also decreased significantly.

China released its 10th edition of COVID-19 control protocols: highlighting vaccination and personal protection.

2023.01.17

The National Health Commission (NHC) made the announcement to lift the requirement of COVID-19 quarantine from Jan8, 2023.

Report from Shanghai community: After reaching a peak in daily new infections, the number of daily new infection has shown a declining trend.

The focus of China's new phase of COVID-19 response is on protecting people's health and preventing severe cases.

Report from Shanghai community: Shanghai is still in a phase of rapid increase of infection.

Implementation of the following policies: more targeted risk area, adjusted quarantine mode, cancellation of PCR requirement, health code and travel code.

Notification: Optimize the process flow in hospitals and improve current medical service

China released a circular on further optimizing the COVID-19 response announcing 10 prevention and control measures in a more science-based and targeted manner.

Scrap PCR testing requirements to enter outdoor public venues such as parks as well as ride public transit.

Twenty adjusted measures were announced, loosening quarantine requirements, close contacts management, and criteria for COVID risk area categorization.

2022.12.29

2022.12.27

2022.12.12

2022.12.09

2022.12.08

2022.12.07

2022.12.05

2022.11.11

Figure S3. The second wave of Omicron in Shanghai


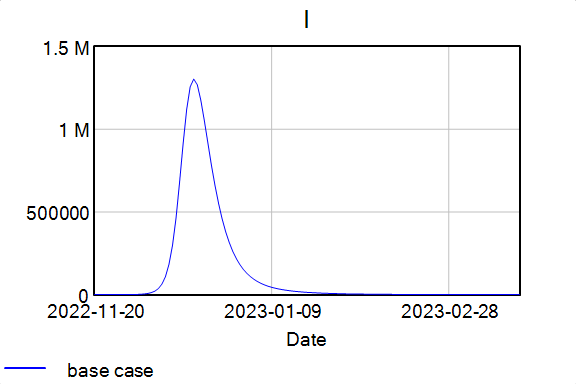


Figure S4 Infected population with symptoms

From figure S4, we can see that the spread of the SARS-COV-2 started to increase from late Nov, entered a soaring period in mid Dec, peaked around Dec 20^th^ 2022 and then underwent a fast decrease. This represents the real situation of the second wave of Omicron in Shanghai at the end of year 2022. Large amount of people infected COVID-19 within short time period of three to four weeks.


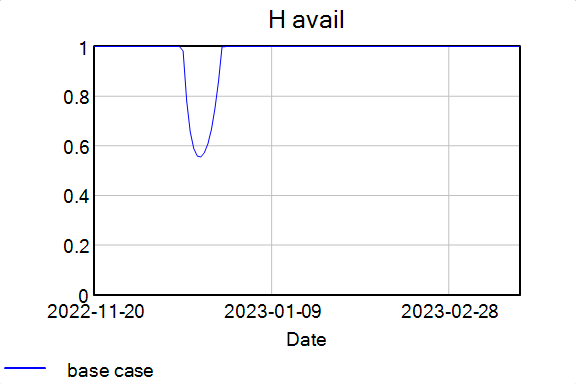

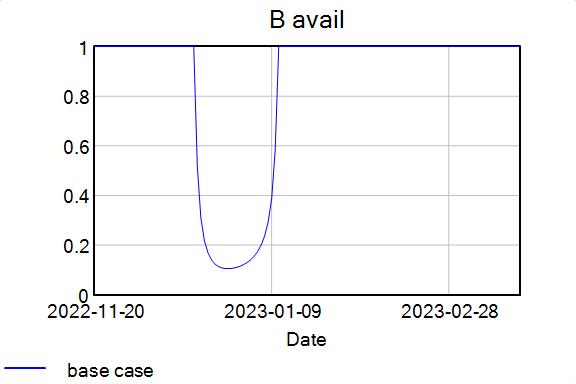


(a) Hospital doctor availability (b) Hospital bed availability

Figure S5 Hospital overwhelm

With fast increase of patients, the hospital in Shanghai had experienced overwhelming pressure. Not enough doctors for the outpatients and the shortage of hospital is even more severe. This simulation does not look for precise replication of the hospital doctor availability and bed availability. It just represent the pattern of what had happened. The model is used as a virtual environment to test the alternative options responding to health emergency.

**3.2 Sensitive test**

| **S1: Test on percent of patients choose to rest at home** | | | |
| --- | --- | --- | --- |
| Variable | Value | Testing | Result |
| a: percent of patients choose to rest at home for recovery | 0.5 | (0.4, 0.6) | Simulation results change in reasonable range, without any change of behavior pattern. |
| 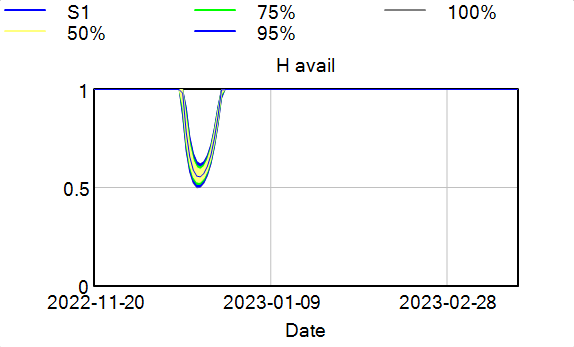 | | 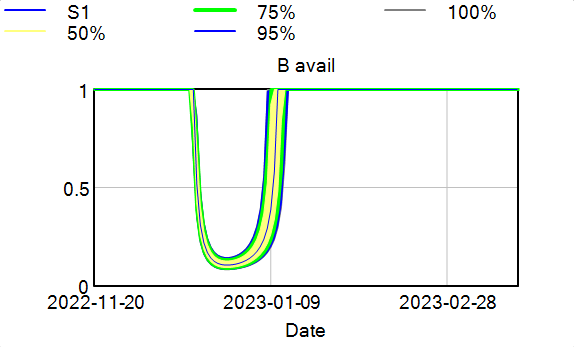 | |
| 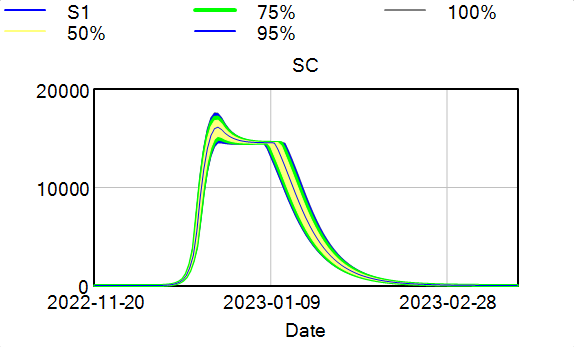 | | 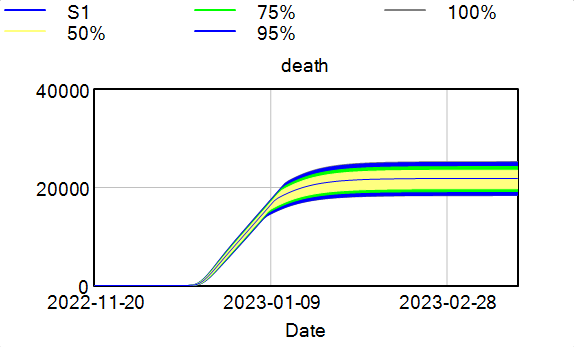 | |

| **S2: Test on percent of patients visited doctor first contact at PHC** | | | |
| --- | --- | --- | --- |
| Variable | Value | Testing | Result |
| d: percent of patients visited doctor first contact at PHC | 0.5 | (0.4, 0.6) | Simulation results change in reasonable range, without any change of behavior pattern. |
| 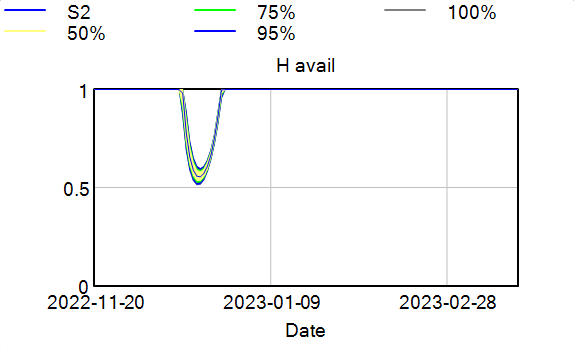 | | 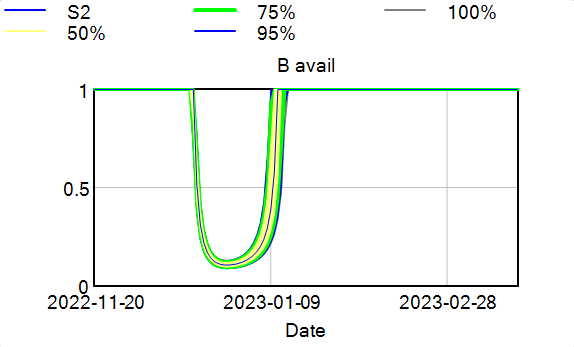 | |
| 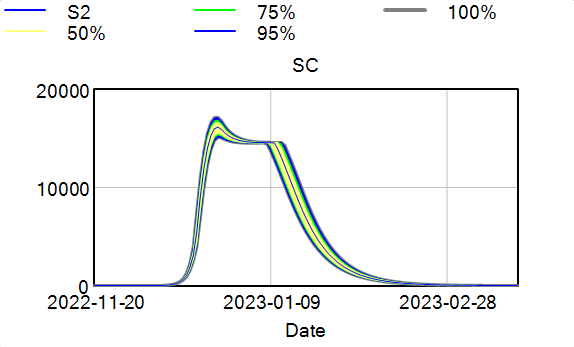 | | 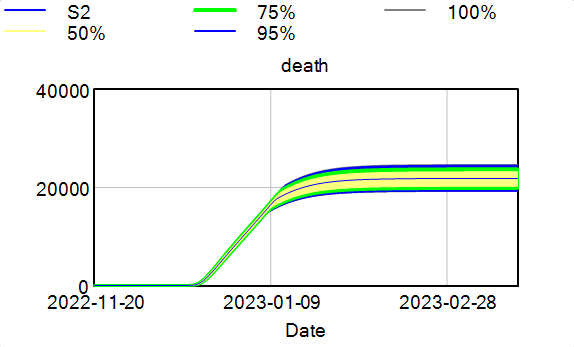 | |

| **S3: Test on percent of treated patients accepted to hospital** | | | |
| --- | --- | --- | --- |
| Variable | Value | Testing | Result |
| e: percent of treated patients accepted to hospital | 0.05 | (0.04, 0.06) | Simulation results change in reasonable range, without any change of behavior pattern. |
| 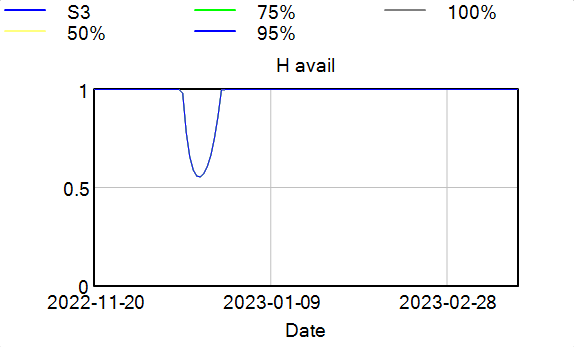 | | 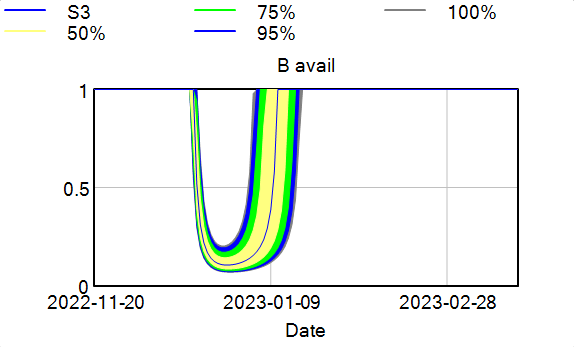 | |
| 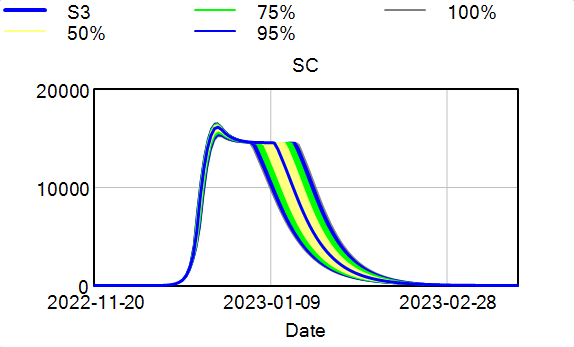 | | 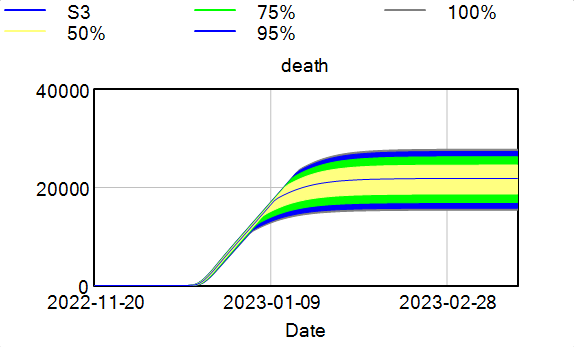 | |

| **S4: Test on percent of patients visited HPC recommended to Hospital** | | | |
| --- | --- | --- | --- |
| Variable | Value | Testing | Result |
| f: percent of patients visited HPC to hospital | 0.1 | (0.05, 0.15) | Simulation results change in reasonable range, without any change of behavior pattern. |
| 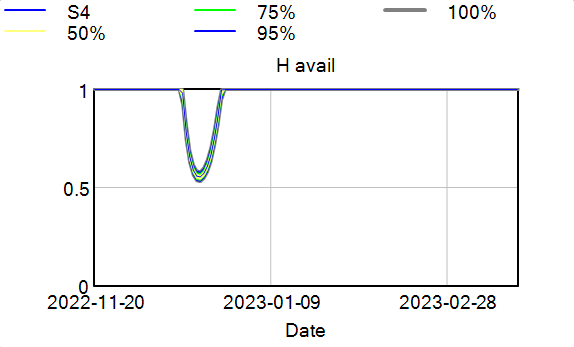 | | 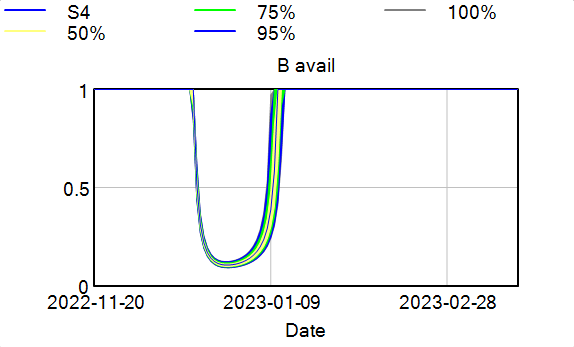 | |
| 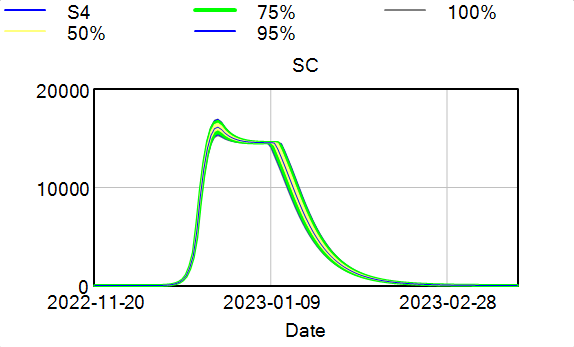 | | 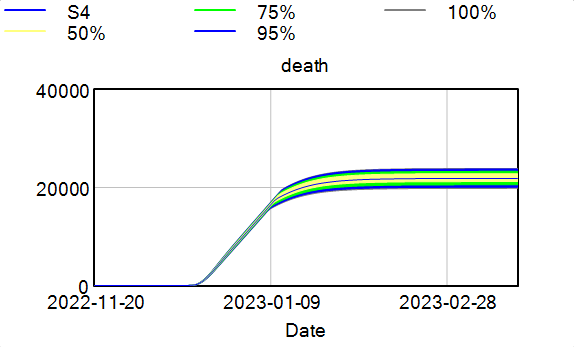 | |

| **S5: Test on percent of patients recover at home get worse** | | | |
| --- | --- | --- | --- |
| Variable | Value | Testing | Result |
| b: percent of patient recover at home at worse | 0.3 | (0.2, 0.4) | Simulation results change in reasonable range, without any change of behavior pattern. |
| 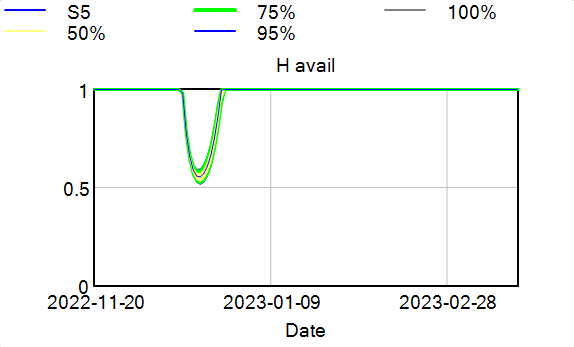 | | 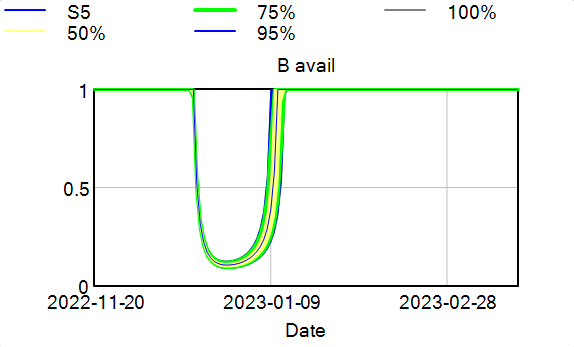 | |
| 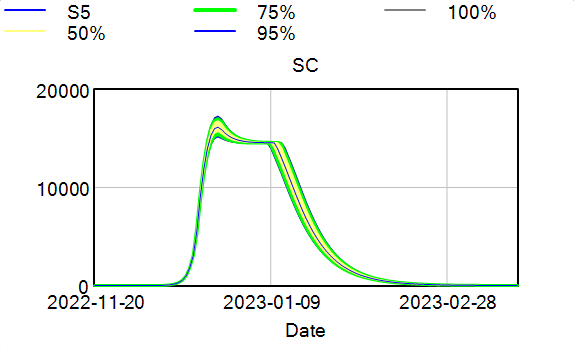 | | 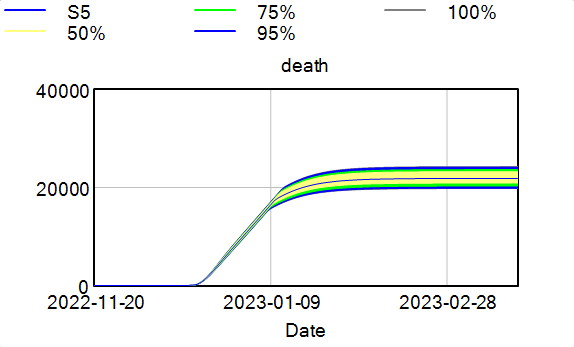 | |

Reference:

1. Piechotta V, Harder T. Waning of COVID-19 vaccine effectiveness: individual and public health risk. The Lancet. 2022;399(10328):887–889.
2. Chemaitelly H, Abu-Raddad LJ. Waning effectiveness of COVID-19 vaccines. The Lancet. 2022;399(10327):771–773.
3. Tang B, Wang X, Li Q, et al. Estimation of the Transmission Risk of the 2019-nCoV and Its Implication for Public Health Interventions. J Clin Med. 2020;9(2):462.
4. Sharp, J. COVID-19: Omicron cases 'doubling every 2-3 days' and expected to be dominant strain within a month; 2022. https://news.sky.com/story/covid-19-omicron-cases-doubling-every-2-3-days-and-expected-to-be-dominant-strain-with-a-month-12490171. Accessed July 27, 2023.
5. Mohsin M, Mahmud S. Omicron SARS-CoV-2 variant of concern: A review on its transmissibility, immune evasion, reinfection, and severity. Medicine. 2022;101(19):e29165.
6. Forward-The Economist. Chinese COVID-19 vaccine has achieved multi-route research and development, and a comprehensive comparison of 5 vaccines that have been on the market; 2022. https://www.qianzhan.com/analyst/detail/220/210607-1bba6e9c.html. Accessed 07 July 2023.
7. Saloman L. Vaccine Protection Against Omicron Wanes Quickly; 2023. https://www.contagionlive.com/view/vaccine-protection-against-omicron-wanes-quickly. Accessed 07 July 2023.
8. Miller K. Omicron's Incubation Period Is Short: Here's Why That Matters; 2022. https://www.prevention.com/health/a38868608/omicron-incubation-period. Accessed 27 July 2023.
9. Masson G. Omicron may have 3-day incubation period, shortest of any variant: 6 things to know; 2021. https://www.beckershospitalreview.com/public-health/omicron-may-have-3-day-incubation-period-shortest-of-any-variant-6-things-to-know.html. Accessed 27 July 2023.
10. Wolters C. How Long Does An Omicron Infection Last in the Body? 2022. [https://www.verywellhealth.com/how-long-does-an-omicron-infection-last-5225306.](https://www.verywellhealth.com/how-long-does-an-omicron-infection-last-5225306.%20Accessed%2027%20July%202023) Accessed 27 July 2023
11. WHO. WHO updates guidelines on treatments for COVID-19. 2023. [https://www.who.int/news/item/10-11-2023-who-updates-guidelines-on-treatments-for-covid-19.](https://www.who.int/news/item/10-11-2023-who-updates-guidelines-on-treatments-for-covid-19.%20)  Accessed 31 Jan 2024
12. Kappler M. People with Omicron much more likely to be asymptomatic, research suggests. 2022 <https://www.healthing.ca/diseases-and-conditions/coronavirus/omicron-asymptomatic-rate/#:~:text=Of%20the%2071%20participants%20who%20tested%20positive%20for,rate%20of%20between%20one%20and%202.4%20per%20cent>. Accessed 31 Jan 2024
